# Novel Insights into the Antagonistic Effects of Losartan against Angiotensin II/AGTR1 Signaling in Glioblastoma Cells

**DOI:** 10.3390/cancers13184555

**Published:** 2021-09-10

**Authors:** Salvatore Panza, Rocco Malivindi, Amanda Caruso, Umberto Russo, Francesca Giordano, Balázs Győrffy, Luca Gelsomino, Francesca De Amicis, Ines Barone, Francesca Luisa Conforti, Cinzia Giordano, Daniela Bonofiglio, Stefania Catalano, Sebastiano Andò

**Affiliations:** 1 Department of Pharmacy, Health and Nutritional Sciences, University of Calabria, 87036 Rende, CS, Italy; salvatore.panza13@gmail.com (S.P.); rocco.malivindi@unical.it (R.M.); amandacaruso22@gmail.com (A.C.); umbrus@live.it (U.R.); francesca.giordano@unical.it (F.G.); luca.gelsomino@unical.it (L.G.); francesca.deamicis@unical.it (F.D.A.); ines.barone@unical.it (I.B.); francescaluisa.conforti@unical.it (F.L.C.); cinzia.giordano@unical.it (C.G.); daniela.bonofiglio@unical.it (D.B.); stefania.catalano@unical.it (S.C.); 2 Centro Sanitario, University of Calabria, 87036 Rende, CS, Italy; 3 Department of Bioinformatics, Semmelweis University, 1094 Budapest, Hungary; gyorffy.balazs@med.semmelweis-univ.hu; 4 Cancer Biomarker Research Group, Research Centre for Natural Sciences, 1117 Budapest, Hungary

**Keywords:** glioblastoma, Angiotensin II, Angiotensin II type I receptor, renin-angiotensin system, aromatase, Losartan, estrogen

## Abstract

**Simple Summary:**

Patients with high-grade glioma (HGG) such as glioblastoma (GBM) who undergo surgical resection with adjuvant therapy have a mean overall survival of 14.6 months and 100% of recurrence. Thus, these disappointing outcomes in terms of glioblastoma life expectancy require seeking novel pharmacological tools, including drug repurposing. In the present study, we identify a novel molecular mechanism through which Losartan antagonizes Angiotensin II (Ang II)/Angiotensin II type I receptor (AGTR1) signaling, overexpressed in GBM cells. For instance, we demonstrate how Losartan drastically inhibits the stimulatory effects of Ang II on aromatase activity and consequently reduces local estrogen production, sustaining cancer progression. Thus, it is reasonable to repurpose Losartan as an adjuvant pharmacological tool to be implemented prospectively in the novel therapeutic strategies adopted in GBM patients.

**Abstract:**

New avenues for glioblastoma therapy are required due to the limited mortality benefit of the current treatments. The renin-angiotensin system (RAS) exhibits local actions and works as a paracrine system in different tissues and tumors, including glioma. The glioblastoma cell lines U-87 MG and T98G overexpresses Angiotensin II (Ang II)/Angiotensin II type I receptor (AGTR1) signaling, which enhances in vitro and in vivo local estrogen production through a direct up-regulation of the aromatase gene promoters p I.f and p I.4. In addition, Ang II/AGTR1 signaling transactivates estrogen receptor-α in a ligand-independent manner through mitogen-activated protein kinase (MAPK) activation. The higher aromatase mRNA expression in patients with glioblastoma was associated with the worst survival prognostic, according to The Cancer Genome Atlas (TCGA). An intrinsic immunosuppressive glioblastoma tumor milieu has been previously documented. We demonstrate how Ang II treatment in glioblastoma cells increases programmed death-ligand 1 (PD-L1) expression reversed by combined exposure to Losartan (LOS) in vitro and in vivo. Our findings highlight how LOS, in addition, antagonizes the previously documented neoangiogenetic, profibrotic, and immunosuppressive effects of Ang II and drastically inhibits its stimulatory effects on local estrogen production, sustaining glioblastoma cell growth. Thus, Losartan may represent an adjuvant pharmacological tool to be repurposed prospectively for glioblastoma treatment.

## 1. Introduction

Gliomas are the most common primary intrinsic tumors in the brain [1,2,3]. For patients with high-grade gliomas such as glioblastoma (GBMs) and patients treated with GTR (gross total resection) adjuvant radiation therapy, and adjuvant chemotherapy with temozolomide (TMZ), the mean survival is 14.6 months [3]. Since the current treatments have given disappointing outcomes related to a significant improvement in the average life expectancy of patients, the incorporation of novel targets and markers into the management of GBM has been suggested [4]. Recently, the known canonical hallmarks of cancer were connected with several biochemical pathways, including those of the renin-angiotensin system (RAS) [5,6,7]. RAS model, which primary action was for a long time limited to the cardiovascular system, has evolved enormously in recent years, letting to discover its connection with the pathways of gliomagenesis [6]. The discovery of the RAS system and receptor in GBM has encouraged the study from a different perspective. For instance, according to transcriptional network analysis, the positive expression of Angiotensin II Receptor Type 1 (AGTR1) and Angiotensin II Receptor Type 2 (AGTR2) have been correlated with different sets of hub genes involved in protumoral function in the C6 glioma cell line, suggesting that both Angiotensin II (Ang II) receptors may represent potential therapeutical targets [8,9]. RAS is also expressed in several tissues such as the liver, kidney, brain, placenta, and ovary [10]. It has been reported that Ang II stimulates estradiol secretion from human placental explant in a dose-time dependent fashion together with a decrease of the total levels of estrogen precursors such as androstenedione and testosterone, addressing how the action of Ang II relies on its stimulatory effect on aromatization step. This effect was blocked by the selective AGTR1 antagonist Losartan [11]. Accordingly, it has also been reported in cattle that short-term exposure to Ang II affects granulosa cell genes involved in estradiol secretion, such as cytochrome P450 aromatase (CYP19) mRNA, determining granulosa cell proliferation, differentiation, and consequently follicle growth [12].

RAS, aromatase, and estrogen production also represent endocrine features of GBM and appear to serve as good prognostic markers. Interestingly, it is worth mentioning that among the three types of receptors involved in estrogen-receptor signaling, such as estrogen receptor-α, estrogen receptor-β and G protein-coupled estrogen receptor (GPER), estrogen receptor-α in terms of mRNA expression and protein content was significantly correlated with malignancy grade of tumor morphology and could be used as a prognostic biomarker of low-grade astrocytoma patients [13].

Whether the stimulatory role of Ang II/AGTR1 signaling on GBM cell growth also involves an induced increase of local estrogen production in glioblastoma sustaining cell growth, as it occurs in other estrogen-responsive tumors, remains to be explored, and this is the issue that we address in the present study.

Accordingly, the logical sequence of the experiments performed in the present study has demonstrated how (1) Ang II/AGTR1 signaling is able to enhance aromatase expression in terms of mRNA, protein content, and enzymatic activity concomitantly with an increase of local estradiol secretion; (2) the above reported up regulatory effect on aromatase expression was due to a direct action of Ang II/AGTR1 signaling on the activation of p I.f and p I.4 aromatase gene promoters; (3) the enhanced local estrogen production induced by Ang II in in vitro assay was enhanced by the combined exposure to an aromatizable steroid such as androstenedione, determining a further increase of glioblastoma cell growth and progression. This highlights the intrinsic capability of Ang II/AGTR1 signaling in modulating intracellular aromatase activity sustaining glioblastoma cell growth. In the same vein, in vivo xenograft revealed how the enhanced tumor growth induced by Ang II was reversed by an inhibitor of aromatase such as Anastrozole; however, the combined treatment of Losartan (LOS) and Anastrazole (ANA) upon angiotensin exposure dramatically reduced tumor volume bringing it below the control value. Similar inhibitory effects were reproduced with the combined treatment of Ang II and LOS. These findings address how LOS encompasses a major comprehensive inhibitory action on tumor growth with respect to ANA.

## 2. Materials and Methods

### 2.1. Reagents and Antibodies

The following reagents and antibodies were used: Angiotensin II (4474-91-3), [1β- 3 H]androst-4-ene3,17-dione (#46033), 17β-estradiol (50-28-2), and anti-AGTR1 (SAB2100073) from Sigma Aldrich (St. Louis, MI, USA); Losartan (#S5067), anti-β-Actin (sc. 69879), and anti-GAPDH (sc. 25778) antibodies from Santa Cruz Biotechnology (Santa Cruz, CA, USA); human anti-STAT3, anti-pSTAT3^Tyr705^, and MAPK/STAT3/pMAPK^Thr202/Tyr204^/pSTAT3 ^Thr705^ from Cell Signaling Technology (Beverly, MA, USA); human anti-Aromatase (MCA2077S) from Bio-Rad Laboratories (Berkeley, CA, USA); Ki-67 (#M724029-2) from Dako Italia Spa (MI, Italy); PD-L1 (#PA5-20343) from Thermo Fisher Scientific, Waltham, MA, USA.

### 2.2. Plasmids

The plasmids pEZX-PL01 containing human aromatase promoter region (pI.f −875/−1) and the 5′-deleted promoter fragment pI.f −674/−1 plasmids were generated by GeneCopeia (Rockville, MD, USA). The plasmid PGL3 containing human aromatase promoter region pI.4 (pI.4 −1004/+14) and the region containing the motif pI.4 GAS mut or pI.4 GRE mut plasmids were provided by Evan R. Simpson (Hudson Institute, Department of Molecular and Translation Science). XETL, a firefly luciferase reporter plasmid bearing estrogen-responsive elements, was supplied by Dr. D. Picard (University of Geneve, Switzerland). HEGO, the wild-type human ERα expression vector, which carries the full-length ERα cDNA fused with the SV40 early promoter and expressed in the pSG5 vector, was furnished from P. Chambon (Institut de Génétique et de Biologie Moléculaire et Cellulaire (IGBMC), Strasbourg, France).

### 2.3. Cell Culture

Human normal glial cells SVG p12, human glioblastoma cell lines U-87 MG and T98G, and cervical carcinoma cell line HeLa were purchased from American Type Culture Collection (Manassas, VA, USA) and authenticated and stored according to supplier’s instructions. Cells were tested for mycoplasma negativity (MycoAlert Mycoplasma Detection Assay, Lonza, Basilea, CH, Swithzerland).

### 2.4. Real-Time RT-PCR Assays

Total RNA from cells was extracted using the TRIzol reagent (Thermo Fisher Scientific). Gene expression was assessed by real-time RT-PCR using SYBR Green Universal PCR Master Mix (Bio-Rad), and the relative gene expression levels were assessed and calculated as previously described [14]. Each sample was normalized on *18S* mRNA content. Primers are listed in Table S1.

### 2.5. Immunofluorescence

Immunofluorescence microscopy analysis was conducted as previously described [15]. Cells were incubated with anti-AGTR1 and anti-aromatase antibodies (4 °C, overnight) and with fluorescein isothiocyanate-conjugated secondary antibody (30 min at RT). As negative control, anti-IgG rabbit and anti-IgG mouse were used. For nuclei detection, 4′,6-Diamidino-2-phenylindole (DAPI; Sigma Aldrich) was used. Fluorescence was photographed with OLYMPUS BX51 microscope (×100 objective) (Tokyo, Japan).

### 2.6. Immunoblot Analysis

Immunoblot assay was assessed as described [16]. Odyssey FC (Licor, Lincoln, NE, USA) and Scion Image laser densitometry scanning program were used, respectively, for acquisition and quantification of each band of interest. The uncropped blots have been reported in Figures S6–S10.

### 2.7. Aromatase Activity Assay

The aromatase activity in subconfluent U-87 MG and T98G cells culture medium was measured using 0.5 µM [1β- 3 H]androst-4-ene3,17-dione as substrate [17] by the tritiated water release assay. The incubations were performed at 37 °C for 5 h under an air/CO_2_ (5%) atmosphere. Data were normalized to mg of protein (fmol/h/mg of protein) and were expressed as picomole/h.

### 2.8. ^[3H]^Thymidine Incorporation

U-87 MG and T98G cells were treated with Ang II with or without the selective Angiotensin II receptor antagonist Losartan for 24 h. For the last 6 h, ^[3H]^Thymidine (1 μCi/mL) was added to the culture medium. Cells, once rinsed with PBS, were washed one time with 10% trichloroacetic acid (TCA) and three times with 5% TCA. Then, cells were lysed by adding 0.1 N NaOH and incubated for 30 min at 37 °C. Scintillation counting was used to determinate ^[3H]^Thymidine incorporation.

### 2.9. Anchorage-Independent Soft Agar Growth Assays

The soft agar anchorage-independent growth assay was assessed as described [18].

### 2.10. Wound-Healing Assays

Cells were plated in 6-well-plate and, after they reached confluence, they were scraped and treated as indicated. The rate of wound healing was quantified after 12 h as previously described [19]. Among three independent experiments, the most representative one was reported in the picture photographed with OLYMPUS BX51 microscope (×10 objective).

### 2.11. Transmigration Assays

Motility was performed as previously described [20].

### 2.12. Transient Transfection Assays

Cells were plated in regular growth medium into 24 well plates and transfected with 1 µg of luciferase reported plasmids and 20 ng of TK Renilla Luciferase plasmid as an internal control using lipofectamine reagent (Thermo Fisher Scientific). After 6 h, treatments were added as indicated, and cells were incubated for 24 h.

Luciferase activity was then measured using the Dual Luciferase Kit (Promega, Milan, Italy) according to the manufacturer’s recommendations. Firefly luciferase activity was normalized to the internal transfection control provided by the Renilla luciferase activity. The normalized relative light unit values obtained from cells treated with vehicle were set as one-fold induction upon which the activity induced by treatments was calculated.

### 2.13. Chromatin Immunoprecipitation Assay

U-87 MG cells were treated with Ang II for 3 h and then subjected to chromatin immunoprecipitation (ChIP). The precleared chromatin was immunoprecipitated with specific anti-STAT3 or anti-c-Jun and anti-polymerase II antibodies and performed as previously described [21]. As a negative control, normal mouse serum IgG was used. Primers are listed in Table S1.

### 2.14. DNA Affinity Precipitation Assay

The binding of nuclear STAT3 and c-Jun to the aromatase promoter I.f and I.4 was assessed in vitro with the DNA Affinity Precipitation Assay (DAPA) as follows. For nuclear protein extract, cells were starved and treated with Ang II (5 µM) for 3 h. A mixture of 100 g of nuclear proteins and 2 g of specific biotinylated DNA probes in 400 µl of buffer D (20 mM HEPES, pH 7.9, 10% glycerol, 50 mM KCl, 0.2 Mm EDTA, 1.5 mM MgCl_2_, 10 M ZnCl_2_, 1 mM dithiothreitol, and 0.25% Triton X-100) was prepared and then incubated on ice for 45 min. Next, 20 µL of streptavidin–agarose beads (Promega) was added, and the samples were incubated under rotation for 2 h at 4 °C. After that, brief centrifugation allowed to collect the agarose bead–protein complexes, which were then washed twice in buffer D. Proteins were uncoupled from DNA probes through the addition of 40 µL of 2× Laemmli’s sample buffer and subsequent heated at 96 °C for 10 min. Centrifugation allowed to remove the beads, and the supernatants were analyzed by immunoblotting. The sequence of GAS oligonucleotide and AP-1 used as probe or the unlabeled competitor is reported in Table S2.

### 2.15. Tumor Xenografts

Female 4–5-week-old athymic nude mice (nu/nu Swiss; ENVIGO RMS, Udine, Italy) were maintained in a 12 h LD (L–light; D–dark) cycle and at constant temperature. A total of 100 μL of U-87 MG cell line (3.0 × 10^6^ cells per mouse) was injected subcutaneously in 100 μL of Matrigel (Corning Costar) of each mouse (*n* = 5 for group). When the mean tumor size reached an average volume of 80–100 mm^3^, the mice were randomly divided, and the treatments were started and delivered as follows: Ang II was administered at a sub-hypertensive dose of 10.8 ng/min through subcutaneously implant osmotic minipumps (DURECT Corporation, Cupertino, CA, USA) after aseptic surgery; LOS was administered via drinking water at a concentration of 66.7 mg/L; ICI 182, 782 was administered weekly at a concentration of 5 mg/mouse via subcutaneous injection. Tumor length and width were measured once a week by caliper measurements, and the volume was calculated according to the following formula: V = (length × width^2^)/2. At the end of the experiment, animals were euthanized and sacrificed; tumors xenografts were dissected, fixed in 4% formalin or frozen in nitrogen, and stored at −80 °C for further analyses. All experiments involving animals and their care were approved by the Ministry of Health (protocol No 737/2020-PR) and were in accordance with the ethics committee of the University of Calabria, Italy.

### 2.16. Histopathologic Analysis

Tissues were embedded in paraffin, fixed and sectioned as described [22].

### 2.17. Immunohistochemical Analysis

Sections (5 μm thick) were mounted on coated glass slides and were deparaffinized and dehydrated (seven to eight serial sections). Tissue sections for immunohistochemical staining were performed as previously described [19] using human anti-Ki-67, anti-aromatase, anti-PD-L1 primary antibodies. Briefly, after incubation overnight with a primary antibody, a specific biotinylated IgG was applied for 1 h at RT, followed by the avidin biotinylated enzyme complex (VECTASTAIN^®^ ABC-HRP KIT, Vector Laboratories, Burlingame, CA, USA). Immunoreactivity was visualized by using 3,3’ diaminobenzidine, DAB (Vector Laboratories). Counterstaining was performed with hematoxylin (Bio-Optica, Milano, Italy). For Ki-67, the slides of tissue tumor samples were evaluated as described [23]. Immunohistochemistry experiments for Aromatase and PD-L1 were evaluated by a pathologist in a blinded manner, and from six to seven serial sections were scored as described in [24] for each sample.

### 2.18. Statistical Analysis

Each datum point represents the mean ± SD of three different experiments. Data were analyzed for statistical significance (*p* < 0.05) using a two-tailed Student’s t-test, performed by GraphPad-Prism7 software program (GraphPad Inc., San Diego, CA, USA). Kaplan–Meier analysis was performed as described [25]. Kaplan–Meier survival graph and hazard ratio with 95% confidence intervals and log-rank *p*-value were calculated and plotted in R using Bioconductor packages. In vivo results were analyzed by Student’s *t*-test or 1-way ANOVA with Bonferroni post hoc testing performed by GraphPad-Prism7 software program (GraphPad Inc., San Diego, CA, USA). All data are reported as mean ± standard deviations (SD), and *p*-value < 0.05 was considered statistically significant.

## 3. Results

### 3.1. AGTR1 Is Overexpressed in GBM Cell Lines

**Figure 1:**
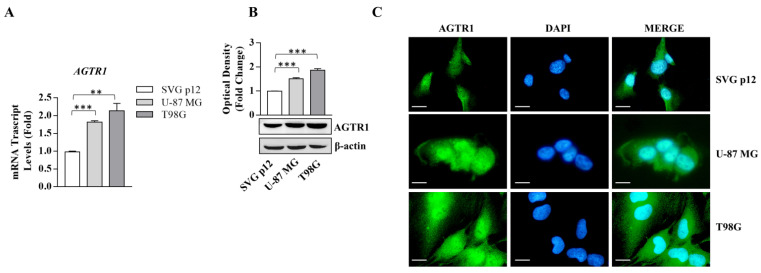
AGTR1 expression in SVG p12 normal glial cells and U-87 MG and T98G glioblastoma cells. (**A**) Real-time RT-PCR for AGTR1 in SVG p12, U-87 MG, and T98G cells; mRNA is shown relative to SVG p12 normal glial cells. (**B**) Immunoblotting showing AGTR1 protein expression. β-actin was used as a loading control. The histograms represent the mean average ± SD of three separate experiments in which band intensities were evaluated in terms of optical density arbitrary unit and expressed as fold change over SVG12 for U-87 MG and T98G. (**C**) Immunofluorescence of AGTR1 in SVG p12, U-87 MG, and T98G cells. DAPI staining for nuclear detection. Scale bars = 5 µm. Original magnification, ×100. Data are expressed as means ± SD of three different experiments, each performed in triplicate. ** *p* < 0.01; *** *p* < 0.001.

AGTR1 expression in terms of mRNA and protein content is significantly upregulated in both U-87 MG and T98G glioblastoma cell lines, displaying different gene mutations [26] compared to normal human glial cells (SVG p12) (Figure 1).

**Figure 2:**
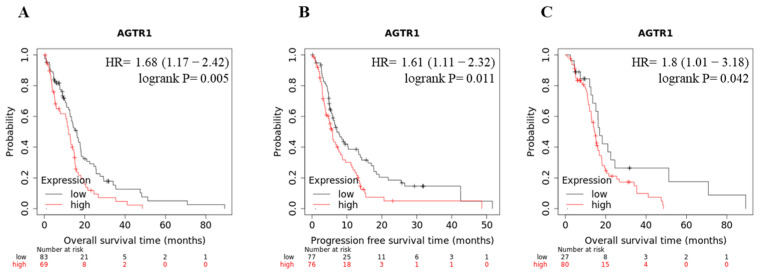
AGTR1 and clinical outcomes in GBM patients. Kaplan–Meier survival analysis relating AGTR1 levels and overall survival (OS) (**A**), progression-free survival (PFS) (**B**), OS in chemotherapy-treated (**C**) GBM patients (TCGA dataset).

The enhanced expression of AGTR1 made us question if AGTR1 could represent a prognostic marker for the clinical outcome of GBM patients. According to the TGCA-GBM dataset, high AGTR1 expression showed significantly lower overall survival (OS) and progression-free survival (PFS) in all GBM patients and shorter overall survival in chemotherapy-treated GBM patients (Figure 2).

**Figure 3:**
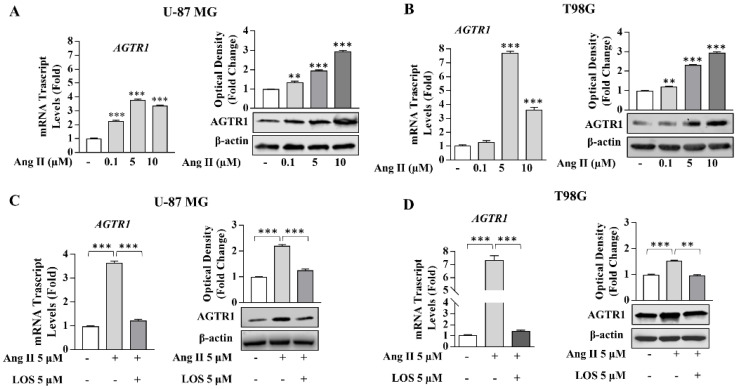
Effects of Ang II receptor antagonist Losartan on Ang II-induced AGTR1 expression in U-87 MG and T98G glioblastoma cells. (**A**,**B**) Real-time RT-PCR and immunoblotting assay for *AGTR1* mRNA and protein expression in U-87 MG and T98G cells treated with vehicle (−) or the Ang II 0.1, 5, and 10 µM for 24 h. β-actin was used as a loading control. The histograms represent the mean average ± SD of three separate experiments in which band intensities were evaluated in terms of optical density arbitrary unit and expressed as fold change over vehicle (−) for Ang II treatment. (**C**,**D**) *AGTR1* mRNA and protein expression in U-87 MG and T98G cells treated with vehicle (−) and Ang II 5 µM alone or in combination with LOS 5 µM for 24 h. The histograms represent the mean average ± SD of three separate experiments in which band intensities were evaluated in terms of optical density arbitrary unit and expressed as fold change over vehicle (−) for Ang II treatment or fold over Ang II for Ang II in combination with LOS. β-actin was used as a loading control. Data represent the mean ± SD of three different experiments, each performed in triplicate. ** *p* < 0.01; *** *p* < 0.001.

Ang II exposure induced upregulation of AGTR1 expression, which was reversed by the selective antagonist Losartan (LOS), highlighting how Ang II/AGTR1 signaling is crucially involved in the positive feedback mechanism regulating its own receptor expression (Figure 3).

Ang II/AGTR1 signaling pathway appeared more active in both glioblastoma cell lines displaying an enhanced phosphorylated status of STAT3 and MAPK in basal condition, which was further increased after Ang II short exposure (Figure S1).

### 3.2. Angiotensin II Increases Growth and Motility in GBM Cells

**Figure 4:**
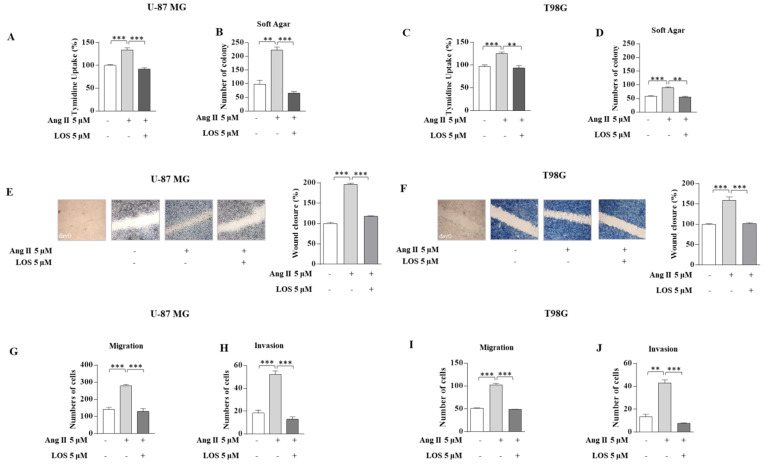
Effects of LOS on Ang II-induced U-87 MG and T98G glioblastoma cell proliferation, migration, and invasiveness. Cell proliferation was determined by the ^[3H]^thymidine (**A**,**C**) and soft agar growth (**B**,**D**) assays in U-87 MG and T98G glioblastoma cells treated with vehicle (−) and Ang II 5 µM alone or in combination with LOS 5 µM for 24 h. (**E**,**F**) Wound-healing assays in U-87 MG and T98G glioblastoma cells treated for 12 h with vehicle (−) and Ang II 5 µM alone or in combination with LOS 5 µM. Images are representative of three independent experiments. The percentage of wound closure has been represented on histograms calculated using ImageJ software version 1.51q. Squares, time 0. Original magnification, ×10. Boyden chamber transmigration (**G**,**I**) and invasion (**H**,**J**) assays in U-87 MG and T98G glioblastoma cells treated with vehicle (−) and Ang II 5 µM alone or in combination with the LOS 5 µM for 12 h. Data represent the mean ± SD of three different experiments, each performed in triplicate. ** *p* < 0.01; *** *p* < 0.001.

Then, we examined the effect of Ang II on the proliferation and motility of GBM cells. Results obtained from thymidine incorporation assays (Figure 4A,C), as well as soft-agar growth assays (Figure 4B,D), show that Ang II significantly enhanced proliferation in U-87 MG and T98G cells. Moreover, Ang II exposure increased cell migration and invasion of GBM cells as evidenced by wound healing, transmigration, and invasion assays, suggesting that this cytokine facilitates the invasive behavior of glioblastoma. The direct involvement of Ang II/AGTR1 signaling in the observed effects was confirmed by their reversion in the presence of LOS (Figure 4).

### 3.3. Angiotensin II Increases Local Estrogen Production in GBM Cells

**Figure 5:**
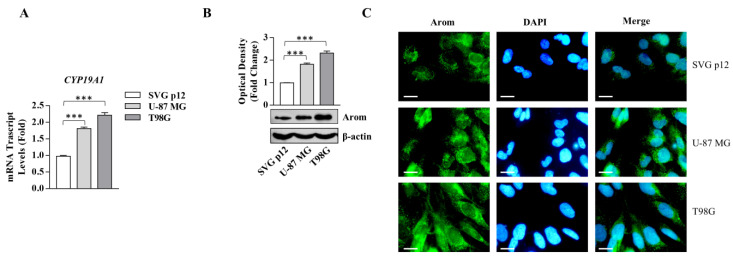
Aromatase expression in SVG p12 normal glial cells and U-87 MG and T98G glioblastoma cells. (**A**) Real-time RT-PCR for *CYP19A1* in SVG p12 normal glial cells, U-87 MG, and T98G glioblastoma cells; mRNA is shown relative to SVG p12 normal glial cells. (**B**) Immunoblotting showing Arom protein expression. β-actin was used as a control for equal loading and transfer. The histograms represent the mean average ± SD of three separate experiments in which band intensities were evaluated in terms of optical density arbitrary unit and expressed as fold change over SVG p12 for U-87 MG and T98G. (**C**) Immunofluorescence of Arom in SVG p12 normal glial cells, U-87 MG, and T98G glioblastoma cells. DAPI staining for nuclear detection. Scale bars = 5 µm. Original magnification, ×100. Data are expressed as means ± SD of three different experiments, each performed in triplicate. *** *p* < 0.001.

Previous findings demonstrated, in a different experimental model, that Ang II is able to enhance local estrogen production, modulating the aromatase activity [11,12]. Here, we tested the effect of Ang II on aromatase activity in GBM cells. First, we evidenced that aromatase (Arom) expression in terms of mRNA and protein content was significantly increased in both glioblastoma cell lines with respect to normal glial cells (Figure 5).

**Figure 6:**
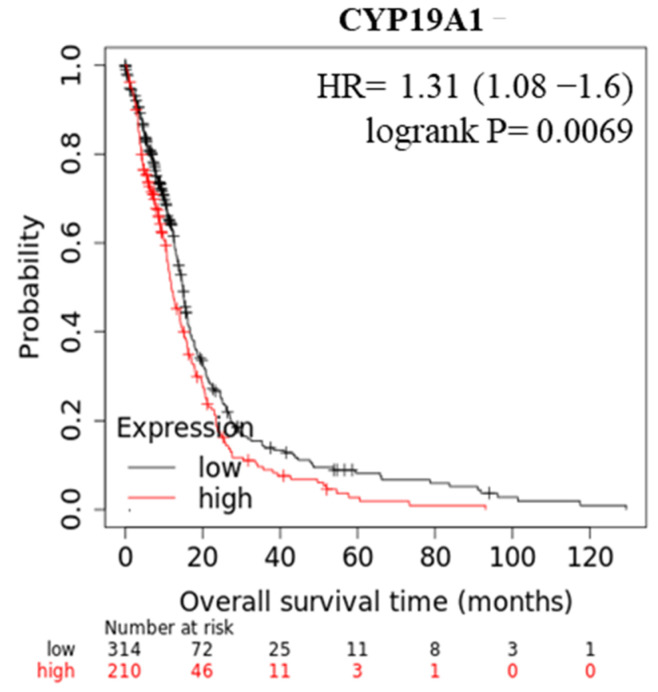
Aromatase and clinical outcomes in GBM patients. Kaplan–Meier survival analysis relating CYP19A1 levels and overall survival (OS) in GBM patients (TCGA dataset).

This brought us to consider if aromatase expression could represent a prognostic marker for the clinical outcome of GBM patients. In the latter concern, our updated Kaplan–Meyer analysis of the TGCA-GBM dataset revealed that patients with high aromatase expression show lower overall survival (Figure 6).

**Figure 7:**
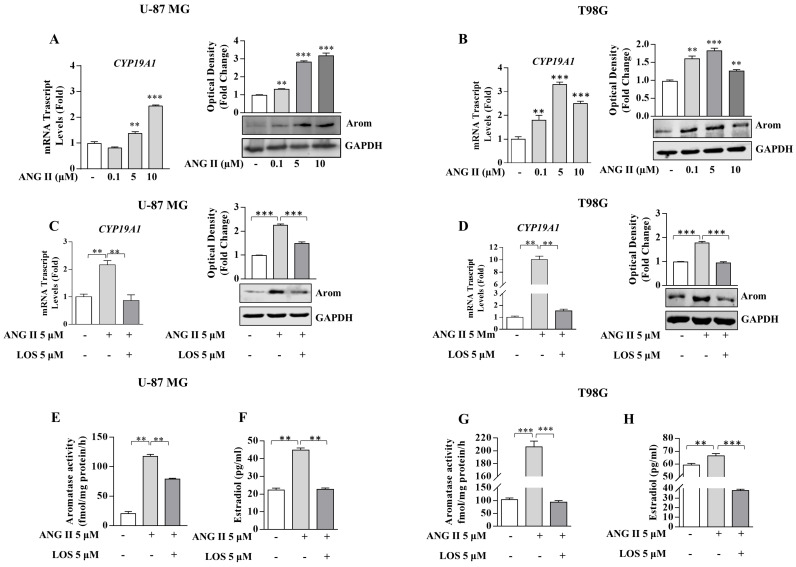
Effects of Ang II and LOS on aromatase expression and activity in U-87 MG and T98G glioblastoma cells. (**A**,**B**) Real-time RT-PCR and immunoblotting assay for *CYP19A1* mRNA and protein expression in U-87 MG and T98G glioblastoma cells, treated with vehicle (−) or the Ang II 0.1, 5, and 10 µM for 24 h. GAPDH was used as a loading control. The histograms represent the mean average ± SD of three separate experiments in which band intensities were evaluated in terms of optical density arbitrary unit and expressed as fold change over vehicle (−) for Ang II treatment. (**C**,**D**) *CYP19A1* mRNA and protein expression in U-87 MG and T98G glioblastoma cells treated with vehicle (−) and Ang II 5 µM alone or in combination with LOS 5 µM for 24 h. The histograms represent the mean average ± SD of three separate experiments in which band intensities were evaluated in terms of optical density arbitrary unit and expressed as fold change over vehicle (−) for Ang II treatment or fold change over Ang II for Ang II in combination with LOS, 5 µM. GAPDH was used as a loading control. (**E**,**G**) Aromatase activity in U-87 MG and T98G glioblastoma cells treated with vehicle (−) and Ang II 5 µM alone or in combination with LOS 5 µM for 24 h. (**F**,**H**) ELISA for Estradiol secretion in U-87 MG and T98G glioblastoma cells treated with vehicle (−) and Ang II 5 µM alone or in combination with LOS 5 µM for 24 h. Data represent the mean ± SD of three different experiments, each performed in triplicate. ** *p* < 0.01; *** *p* < 0.001.

It is important to remark how Ang II exposure enhanced in both GBM cell lines the expression of aromatase in terms of mRNA, protein content and enzymatic activity. All these events were reversed in the presence of LOS (Figure 7). Furthermore, AGTR1 knockdown unequivocally abrogated the up-regulatory effects of Ang II on aromatase expression, evidencing the specificity of Ang II/AGTR1 signaling in upregulating aromatase expression (data not shown).

**Figure 8:**
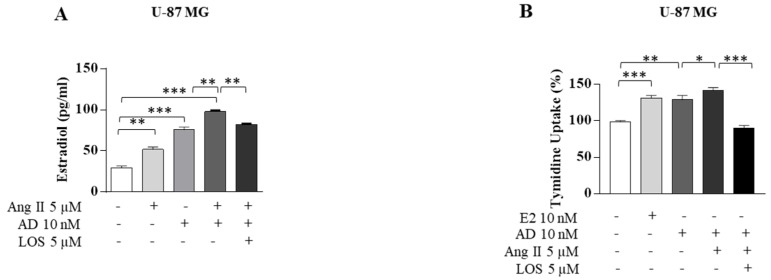
Ang II potentiates the estradiol levels produced by an aromatizable steroid androst-4-ene-3,17-dione in U-87 MG glioblastoma cells. (**A**) ELISA for Estradiol secretion treated with vehicle (−), Ang II 5 µM, androst-4-ene-3,17-dione (AD 10 nM), Ang II 5 µM plus AD 10 nM, and Ang II 5 µM plus AD 10 nM plus LOS 5 µM for 24 h. (**B**) Cell proliferation was determined by the ^[3H]^thymidine assay in U-87 MG cells treated with vehicle (−), estradiol (E2 10 nM), Ang II 5 µM, AD 10 nM, Ang II 5 µM plus AD 10 nM, and Ang II 5 µM plus AD 10 nM plus LOS 5 µM for 24 h. Data are expressed as means ± SD of three different experiments, each performed in triplicate. * *p* < 0.05; ** *p* < 0.01; *** *p* < 0.001.

The biological correlate of the up-regulatory effect induced by Ang II on aromatase expression was represented by an enhanced production of estradiol secreted by both GBM cells upon Ang II exposure. It is interesting to observe how estradiol produced by both tested glioblastoma cell lines in the presence of an aromatizable steroid such as androstenedione was significantly increased by the combined exposure to both Ang II and androstenedione, confirming again the intrinsic properties of Ang II/AGTR1 signaling to stimulate the intracellular aromatase enzymatic activity (Figure 8A). In the presence of either Ang II or androstenedione, GBM cell proliferation increased and appeared markedly enhanced by the combined treatment (Figure 8B). These events were abrogated in the presence of LOS (Figure 8A,B).

### 3.4. Ang II/AGTR1 Signaling Activates Aromatase Promoter pI.f and pI.4

**Figure 9:**
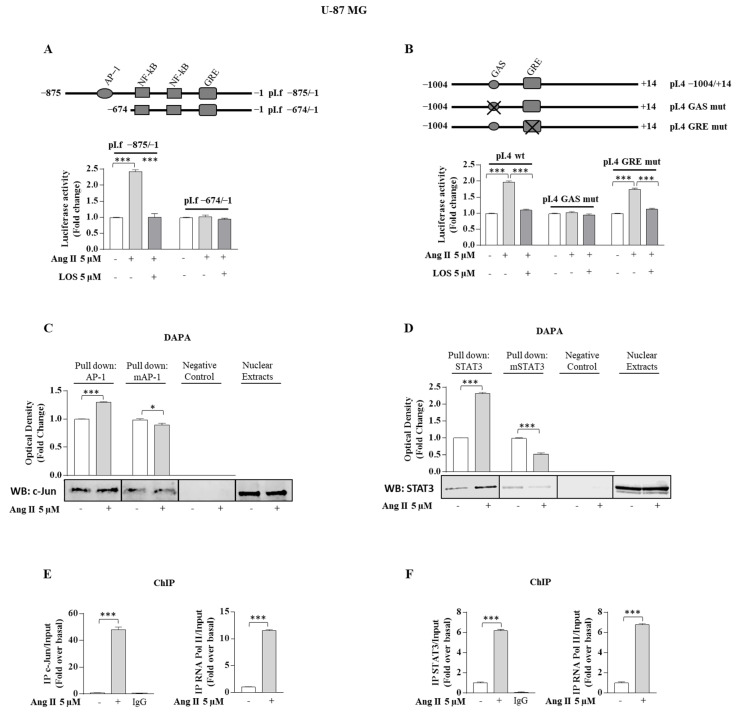
Aromatase promoters pI.f/I.4 in U-87 MG glioblastoma cells are stimulated upon Ang II exposure. (**A**,**B**) Upper panel, schematic map of the pI.f and pI.4 aromatase constructs. U-87 MG glioblastoma cells were transiently transfected with the reported constructs and treated with vehicle (−) or Ang II 5 µM, alone or in combination with LOS 5 µM for 24 h. (**C**,**D**) U-87 MG glioblastoma cells were treated in the presence of vehicle (−) or Ang II 5 µM for 3 h. Nuclear extracts were incubated with a biotinylated oligonucleotide containing the AP-1 or AP-1 mutant site in the aromatase promoters pI.f (left panel) or with a biotinylated oligonucleotide containing the STAT3 or STAT3 mutant site in the aromatase promoters pI.4 (right panel) and subjected to DNA affinity precipitation assay. Specifically bound proteins were subjected to Western blotting analysis. The specificity of the binding was tested by loading the unbound fraction (Negative Control). U-87 MG glioblastoma cells nuclear extracts were used as positive control. The histograms represent the mean average ± SD of three separate experiments in which band intensities were evaluated in terms of optical density arbitrary unit and expressed as fold change over vehicle (−) for Ang II treatment. (**E**,**F**) U-87 MG glioblastoma cells were treated in the presence of vehicle (−) or Ang II 5 µM for 3 h, then cross-linked with formaldehyde and lysed. The precleared chromatin was immunoprecipitated with anti-c-Jun or anti-RNA Pol II (left panel) or with anti-STAT3 and anti-RNA Pol II (the right panel). The 5′flanking sequence of the *CYP19A1* gene was detected by real-time PCR with specific primers to amplify aromatase promoter sequence, including the AP-1 and STAT3 sites. Input DNA was amplified as loading controls. Data are expressed as means ± SD of three different experiments, each performed in triplicate. * *p* < 0.05; *** *p* < 0.001.

In order to ascertain if Ang II/AGTR1 signaling was able to directly affect aromatase at transcriptional levels, we evaluated its effect on the promoter aromatase gene activity. It is well known how the aromatase gene exhibits tissue-specific promoters correspondent to different 5′ untranslated first exon (exon I), spliced upstream of a common translation start site in the coding region (exon II). It is uncertain if aromatase promoters, expressed and working in glioma, are the same as those working in normal human glia. Recent findings have reported that GBM cell line T98G displays the same aromatase promoter transcripts expressed in the human temporal cortex (pII, pI.3, pI.f, pI.4) [27]. However, the two latter promoters exhibit the same pattern of expression in GBM cells and in the temporal cortex, so we focused our attention on the effect of Ang II on the aromatase pI.f and pI.4 promoters to investigate its direct capability to modulate aromatase gene expression. Upon Ang II exposure, a significant increase of both promoters’ activity was observed (Figure 9A). The pI.f promoter region, containing the AP-1 motif, has been considered to be potentially involved in mediating the transactivation mechanisms exerted by Ang II, being AP-1 motif an effector of Ang II/AGTR1 signaling. Indeed, the deletion of the region that contains the AP-1 motif (−875/−674 region) abrogated the pI.f promoter activation upon Ang II exposure (Figure 9A). To further investigate the functional importance of the AP-1 motif in the pI.f promoter, we carried out DAPA assay by using a double-stranded oligonucleotide. Endogenous c-Jun protein, which is a component of either AP-1 homodimer c-Jun/c-Jun or AP-1 heterodimer c-Jun/c-Fos, was found associated with the putative consensus oligonucleotide following Ang II administration. A mutant oligonucleotide abolished c-Jun binding, confirming the specificity of the site identified in vitro (Figure 9C). Furthermore, to demonstrate the involvement of the AP-1 site in Ang II-mediated upregulation of aromatase pI.f, ChiP assay was performed using specific antibodies against c-Jun and Polymerase II; protein chromatin complexes were immunoprecipitated in human culture maintained with or without Ang II for 3 h. The precipitated DNA was then quantified using real-time PCR with primers spanning the AP-1 binding element in the aromatase pI.f promoter region. c-Jun recruitment was significantly upregulated upon Ang II treatment in the U-87 MG cell line. These results were correlated with a high association of RNA polymerase II to the aromatase regulatory region (Figure 9E). To identify the region within the aromatase promoter pI.4 that is functionally important for transcriptional regulation by Ang II, transient transfection experiments were performed by using two constructs pI.4 mutants (pI.4 GAS mut and pI.4 GRE mut). Disruption of the putative GAS consensus site drastically reduced the up-regulatory effect on pI.4 promoter activity upon Ang II exposure, confirming that the GAS motif is a critical sequence involved in the promoter activation upon Ang II exposure (Figure 9B). Similarly, the DAPA assay confirmed the functional importance of the GAS sequence, evidencing how the endogenous STAT3 was found associated with the double-stranded oligonucleotide exhibiting the further consensus sequence upon Ang II exposure. The latter event was completely abrogated in the presence of a mutant oligonucleotide (Figure 9D). ChiP assay, using specific antibodies against STAT3 and Polymerase II, revealed a DNA that, when quantified using real-time PCR with primers against the STAT3 binding elements, showed an up-regulation of STAT3 recruitment upon Ang II treatment in U-87 MG cell line (Figure 9F).

### 3.5. Evidence That Ang II Transactivates Estrogen Receptor-α in GBM Cell Lines

Taking into account that unliganded estrogen receptor-α is an effector of MAPK signaling and that Ang II is able to activate the MAPK pathway via Janus-kinase, we investigated whether Ang II is able to induce transactivation of the endogenous estrogen receptor-α in GBM cells. Thus, we transfected U-87 MG cells with XETL, and we found that Ang II significantly increased XETL reporter activity. To further support the direct involvement of Ang II on estrogen receptor-α transactivation, we co-transfected estrogen receptor-negative HeLa cells with HEGO and XETL, and in the same experimental conditions, we obtained the same results (Figure S2). Remarkably, LOS or the pure antiestrogen ICI 182, 780 (ICI), or MAPK inhibitor PD98059 (PD) were able to efficiently antagonize the stimulatory effect of Ang II on estrogen receptor-α regulated transactivation in both U-87 MG and HeLa cells, evidencing the intrinsic property of Ang II/AGTR1 signaling to transactivate estrogen receptor-α independently on cell type-specificity. Further evidence of the capability of Ang II to transactivate the estrogen receptor-α was provided by the strong upregulation of the expression of classical estrogen-dependent genes, such as *TFF1*, *CCND1,* and *CATSD* upon Ang II exposure, which was reversed by the addition of either LOS or ICI (Figure S3).

### 3.6. Ang II/AGTR1 Signaling Enhances Intrinsic Immunosuppressive Effect of GBM Cells through PD-L1 Secretion

It has been previously evidenced how glioblastoma sustains an immunotolerant milieu. One of the checkpoint inhibitors, generally expressed in several cancer cells, including cells of glioma, is the programed death-ligand 1 (PD-L1). Thus, we investigated how different treatments in our experimental models could influence PD-L1 expression in glioblastoma cells. We found a marked increase of PD-L1 expression in U-87 MG cells treated with Ang II, which was partially reversed by either LOS or ICI (Figure S4A).

### 3.7. Losartan Treatment Inhibits Tumor Growth in U-87 MG Xenograft

**Figure 10:**
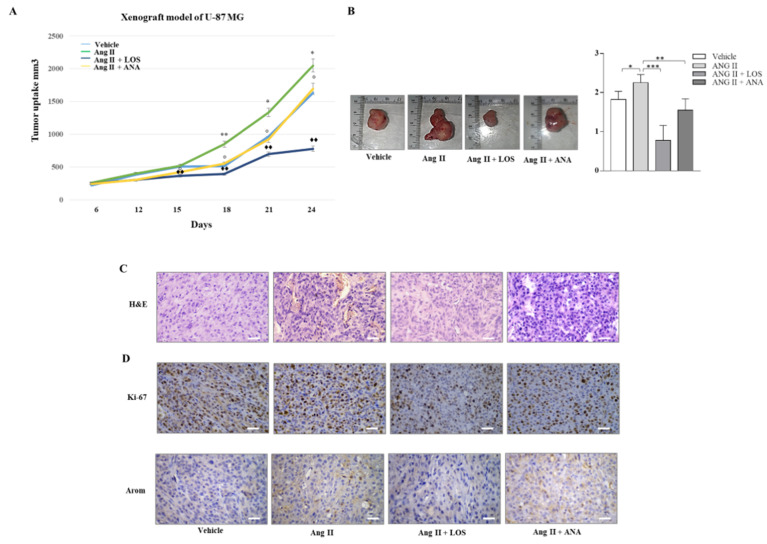
LOS Inhibits Ang II-induced tumor growth of U-87 MG xenografts. (**A**) U-87 MG glioblastoma cells were injected subcutaneously in female nude mice (five mice per group) and then treated with vehicle (−), Ang II alone, or in combination with LOS or ANA. Relative tumor volume (RTV) was calculated by the following formula: RTV = (Vx/V1) where Vx is the tumor volume on day x and V1 is the tumor volume at initiation of treatment (day 0). y-axis: means ± SD of the RTV. (**B**) Images of representative individual tumors and average tumor weight from vehicle (−), Ang II alone, or in combination with LOS or ANA. (**C**) Hematoxylin and eosin (H&E) staining of tumor sections from vehicle (−), LOS and Ang II alone, or in combination with LOS or ANA. (**D**) Immunohistochemical analysis in U-87 MG xenograft tumor of Ki-67, Arom, upon Ang II treatment w/o LOS or w/o ANA and both LOS and ANA. Scale bars = 12.5 μm. * *p* < 0.05; ** *p* < 0.01; *** *p* < 0.001.

To investigate the effect of Ang II/AGTR1 signaling on cell growth and progression, we performed xenograft experiments. To this aim, we injected U-87 MG cell line in the interscapular region of female nude mice and monitored tumor growth after administration of vehicle, Ang II, Ang II with LOS, and Ang II with an inhibitor of aromatase Anastrozole (ANA). The tumor was detected six days after injection, and tumor size was monitored every three days until day 21. This administration was tolerated because no changes in body weight or in food and water consumption were observed, along with no evidence of reduced motor function. In addition, a non-significant difference in the mean weight or in the histological features in the liver and kidney after sacrifice was observed between the vehicle and treated mice, indicating a lack of toxic effects. Treatment with Ang II increased tumor growth significantly with respect to the vehicle group, which was reversed by ANA. However, it was extremely impressive that treatment with LOS drastically antagonized the effect of Ang II on tumor growth and reduced glioblastoma tumor volume to a size corresponding to almost half of that of the control (Figure 10A,B). A significant increase of Ki-67 expression, a well-known proliferation marker, was observed upon Ang II exposure, which was attenuated by the combined treatment with ANA but drastically reduced upon the combined exposure with LOS (Figure 10C, Table 1). Aromatase appears clearly upregulated upon Ang II, still augmented upon Ang II and ANA and definitely reduced upon Ang II and LOS (Figure 10D). Assuming that estrogen receptor-α signaling stimulates glioblastoma cell growth just as it does in other estrogen-sensitive cancer, we questioned to which extent the intrinsic capability of Ang II to transactivate estrogen receptor-α may influence its stimulatory effects on GBM cell growth.

Thus, in a separate set of xenograft experiments, we monitored tumor growth after administration of Ang II and Ang II with the pure antiestrogen ICI (Figure S5). It’s worth observing how tumor growth obtained upon Ang II treatment was reversed by ICI, definitely placing the glioblastoma among the estrogen-responsive tumors. Then, we observed upon Ang II treatment as expected an increased expression of aromatase (data no show) and of Ki-67, which was decreased by ICI (Figure S5, Table S3).

Immunostaining in xenograft tumors tissue confirmed how Ang II exposure enhanced the expression of the immune-checkpoint inhibitor PD-L1 in glioblastoma cells drastically reversed by LOS (Figure S4B).

## 4. Discussion

It was demonstrated that Angiotensin II (Ang II) is not able to cross the blood–brain barrier (BBB), and consequently, its content in the brain has been reasonably considered to not come from the systemic renin-angiotensin system (RAS) [28]. This has been subsequently sustained by the evidence that essential substrates and enzymes involved in the synthesis of bioactive Angiotensin peptide can be produced locally in the brain but not found in a single cell and separately from the peripheral system [29]. Thus, a complete network of intracellular interaction was required to obtain its bioactive neuropeptide able to sustain proliferative signaling, angiogenesis, evading growth suppression, resisting apoptosis, and regulating cellular invasion and metastasis [30,31]. Ang II/AGTR1 is expressed in tumors such as the GBM, and it is associated with tumor growth and with a more aggressive tumor phenotype [32]. Previously, it has been reported in different experimental models how Ang II enhances estradiol production through a stimulatory effect on the aromatization step of steroid precursors [11,12]. In addition, aromatase has been assessed by RT-PCR and immunohistochemistry in the rat and in two human glioblastoma cells (T98G and U373), displaying a cytoplasmatic pattern of immunoreactivity [27]. In the present study, we evidenced how aromatase in GBM cells is highly expressed and, in analogy to its role in other estrogen-sensitive cancers, it can be reasonably assumed that this enzyme is responsible for local estradiol production stimulating cell growth and progression. Estrogen secretion by GBM cells is well correlated with aromatase enzymatic activity. A previous study reported that intratumoral estradiol concentrations in 36 biopsies patients with astrocytoma progressively increase in a positive manner related to grade malignancy [13]. The same authors found a positive correlation between E2 concentration and aromatase when expressed in terms of the relative unit of *CYP19A1* mRNA expression, which was also related to patients’ dead risk. Finally, the existence of a link between patient overall survival and mRNA aromatase units was reported, as shown in the present study. In the above-mentioned study [13], it was described that a decrease of estrogen receptor-α expression inversely correlated with estradiol concentration. We may reasonably explain the latter finding with the fact that intratumoral estradiol concentrations down-regulate the expression of estrogen receptor-α in terms of mRNA and protein content as a classical biological feature of the occurred estrogen receptor-α wild type functional transactivation [33,34]. The data of Jiménez research work [13] appeared to not fit with those of Honikl and coworkers [35], who reported how in 60 glioblastoma patients, the increase of aromatase is concomitant with a longer survival even though the intratumoral estradiol concentrations are not documented. In addition, the latter study reported how the administration of estrogen at supraphysiological doses ranging from 10 to 50 µM dramatically reduced glioblastoma cell viability. Therefore, we reasonably assumed that these doses of estrogen, being up to five thousand times as much higher than the physiological ones used in the present study, produced a cytotoxic effect similar to that previously documented in breast cancer [36] and mentioned by the author himself.

Ang II/AGTR1 signaling enhances local estrogen production in GBM through the induced upregulation of aromatase gene expression. This gives more emphasis to the common involvement of AGTR1 and aromatase as a prognostic index of the clinical outcome, both being significantly linked to the overall survival of GBM patients. 

Additionally, from the present findings, the intrinsic capability of Ang II to transactivate estrogen receptor-α emerged and enhanced the expression of the classical estrogen-dependent genes. This allowed us to investigate how the pure antiestrogen ICI treatment may modulate the Ang II effect in GBM cell growth. The evidence that Losartan (LOS) inhibits the stimulatory effect of Ang II in glioblastoma cell proliferation to a significantly greater extent than ICI as revealed by the Ki-67 immunostaining score indicates that such inhibitory effect does not come exclusively from the concomitant downregulation of estrogen receptor-α signaling, but depends on additional events mostly related to the previous described antiangiogenetic and antifibrotic effect of LOS [37,38,39,40,41]. For instance, LOS has been found to reduce stromal collagen I and hyaluronan content impairing ECM organization, thus decreasing mechanical forces that contribute to the intratumoral solid stress. The latter event lowers the compression of blood vessels, improving the delivery of drugs and oxygen. The molecular background of all the described events is the LOS inhibitory interference with transforming growth factor-beta 1 (TGF-β1) signaling taking part in different cellular signaling pathways affecting tumor stroma as well as cellular behavior, including migration, proliferation, differentiation, apoptosis, and particularly epithelial–mesenchymal transition (EMT) in glioblastoma [38,39,40,41,42,43,44]. Based on our present findings, we formulated the idea that the inhibitory effect of LOS on the EMT process in GBM cells may also be indirectly reinforced by its inhibitory action on local estrogen production [45,46]. Indeed, estradiol not only stimulates glioblastoma cell proliferation well-fitting with our present data [47] but also induces a change in cell morphology displaying an elongated cell phenotype correlated with actin filaments rearrangements, together with an increase of cell migration, invasion, and upregulation of EMT markers such as Vimentin and N-Cadherin in GBM cell lines [47,48,49,50].

Finally, in recent years, it has been evidenced how glioblastoma cells display an intrinsic immunosuppressive tumor milieu. Tumor cells can weaken anti-tumor immunity by activating the so-called immune checkpoint molecules (ICs) [51]. Programed cell death ligand-1 (PD-L1), secreted by tumor cells, combined with programmed cell death-1 (PD-1) located on the surface of activated T-cell, macrophages, etc., in the tumor microenvironment, promotes immune tolerance sustaining tumor immune escape, growth, and progression [52]. Various cancers display an enhanced PD-L1 expression associated with unfavorable prognosis [53,54,55,56,57,58]. Thus, targeting PD-1/PD-L1 has been the goal of the novel immunotherapeutic strategies, providing a remarkable prognosis improvement in different solid tumors previously hard to be treated [59,60,61,62,63,64]. Interestingly, meta-analysis has demonstrated how PD-L1 expression in glioblastoma may have a clinical and prognostic significance [65]. In the present study, we demonstrated how Ang II exposure concomitantly with a clear increase of aromatase expression, local estrogen production, and intrinsic capability to transactivate estrogen receptor-α, upregulated PD-L1 expression. It was well demonstrated that STAT3 signaling is a regulator of PD-L1 expression in mouse models and in cancer cell lines. Therefore, with STAT3 being a crucial mediator of Ang II/AGTR1 signaling, it is plausible that this signaling stimulates PD-L1 expression. However, it’s worth mentioning that sequence analysis of human PD-L1 promoter also revealed estrogen-responsive elements (EREs) sustaining estrogen responsiveness. This recalls previous findings showing how in vitro estradiol treatment upregulated PD-L1 expression on CD8+ T-cells from CX-service and on estrogen receptor-α positive cancer cell lines. [66,67]. Finally, the up-regulatory effect of Ang II on PD-L1 expression appeared partially reversed upon LOS and the antiestrogen ICI in terms of mRNA and protein content. Immunostaining in vivo confirmed the marked upregulation of PD-L1 displayed by tumor cells upon Ang II exposure which was reversed by ICI and by LOS to a greater extent. Our results concerning the up-regulatory role of Ang II/AGTR1 signaling on PD-L1 expression sustaining immune tolerance in glioblastoma cells, even though extremely promising, need to be corroborated by further studies related to the immunosuppressive effects of angiotensin on the tumor microenvironment by performing a more proper experimental model that represents orthotopic implantation of glioblastoma cells in syngeneic mice.

## 5. Conclusions

In conclusion, from our findings, it emerges how the RAS system in glioblastoma cells potentiates local estrogen production through an “intracrine” integrated system sustaining tumor progression. Accordingly, it has been evidenced how in the broad spectrum of biological actions performed by Losartan as a classical RAS antagonist, it assumes its capability to inhibit aromatase activity and the consequent local estrogen production as an important stimulatory component of tumor growth and progression. The last action, together with those previously documented on tumor stroma and on immune features of tumor microenvironment, make us consider Losartan as an adjuvant pharmacologic tool to be prospectively repurposed for the novel therapeutic strategies adopted in glioblastoma patients.

## Figures and Tables

**Table 1:** Immunostaining scores (Allred score median) of Ki-67 in U-87 MG xenograft treated with Ang II alone or in combination with LOS or ANA or ICI.

Antibody	Vehicle	Ang II	Ang II + LOS	Ang II + ANA
Ki-67	6	7	3 **	3 *

Note: Immunostained slides scores as follows: Total score = Proposition score + Intensity score (range 0–8); * *p* < 0.005 (one-way ANOVA test) vehicle versus treated; ** *p* < 0.001 (one-way ANOVA test) vehicle versus treated.

## Data Availability

The data presented and the data not shown in this study are available on request from the corresponding author. The data are not publicly available due to privacy reasons.

## Supplementary Materials

The following are available online at https://www.mdpi.com/article/10.3390/cancers13184555/s1, Figure S1: Angiotensin II signaling activation in normal glial and GBM cells, Figure S2: Transactivation of ERα in U-87 MG cells and HeLa cells, Figure S3: Effects of Angiotensin II receptor antagonist Losartan and ICI 182, 780, on ERα target genes expression in U-87 MG cells, Figure S4: Effect of Angiotensin, Losartan and ICI on PD-L1 expression in glioblastoma cells, in vitro and in vivo, Figure S5: ICI 182, 780 downregulates Ang II-induced tumor growth of U-87MG xenograft, Figures S6–S10: Uncropped Western blots from primary figures are shown, Table S1: Oligonucleotide primers for real-time PCR assays, Table S2: Oligonucleotide primers for DAPA assays.
