# Supplementary material for: Novel Insights into the Antagonistic Effects of Losartan against Angiotensin II/AGTR1 Signaling in Glioblastoma Cells
Source: Cancers (Basel). 2021 Sep 10;13(18):4555. doi: 10.3390/cancers13184555 (PMC8469998; doi:10.3390/cancers13184555)
Supplement: Supplementary file 1 [file cancers-13-04555-s001.zip › Supplementary PDF/Figure S1.pdf]

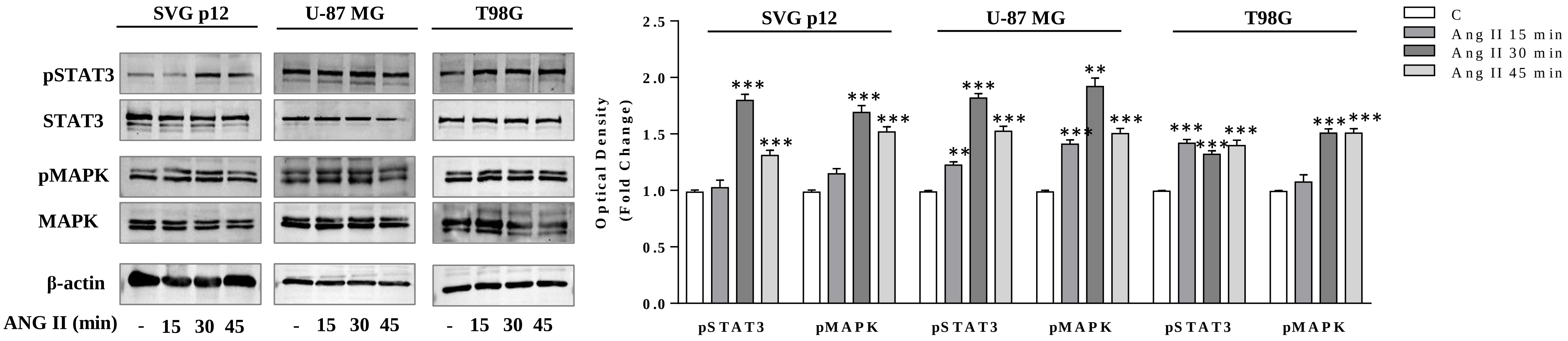

Figure S1. Angiotensin II signaling activation in normal glial and GBM cells. Immunoblotting of phosphorylated signal transducer and activators of transcription (pSTAT)3/phosphorylated mitogen-activated protein kinase (pMAPK) and total nonphosphorylated proteins from normal glial cell (SVG p12) and glioblastoma cells (U-87 MG and T98G) treated with vehicle (-) or 5 µM Angiotensin II (ANG II). β-Actin was used as loading control. The histograms represent the mean average ± S.D. of three separate experiments in which band intensities were evaluated in terms of optical density arbitrary unit and expressed as fold change over vehicle (-) for Ang II treatment.
