# Supplementary material for: Novel Insights into the Antagonistic Effects of Losartan against Angiotensin II/AGTR1 Signaling in Glioblastoma Cells
Source: Cancers (Basel). 2021 Sep 10;13(18):4555. doi: 10.3390/cancers13184555 (PMC8469998; doi:10.3390/cancers13184555)
Supplement: Supplementary file 1 [file cancers-13-04555-s001.zip › Supplementary PDF/Figure S3.pdf]

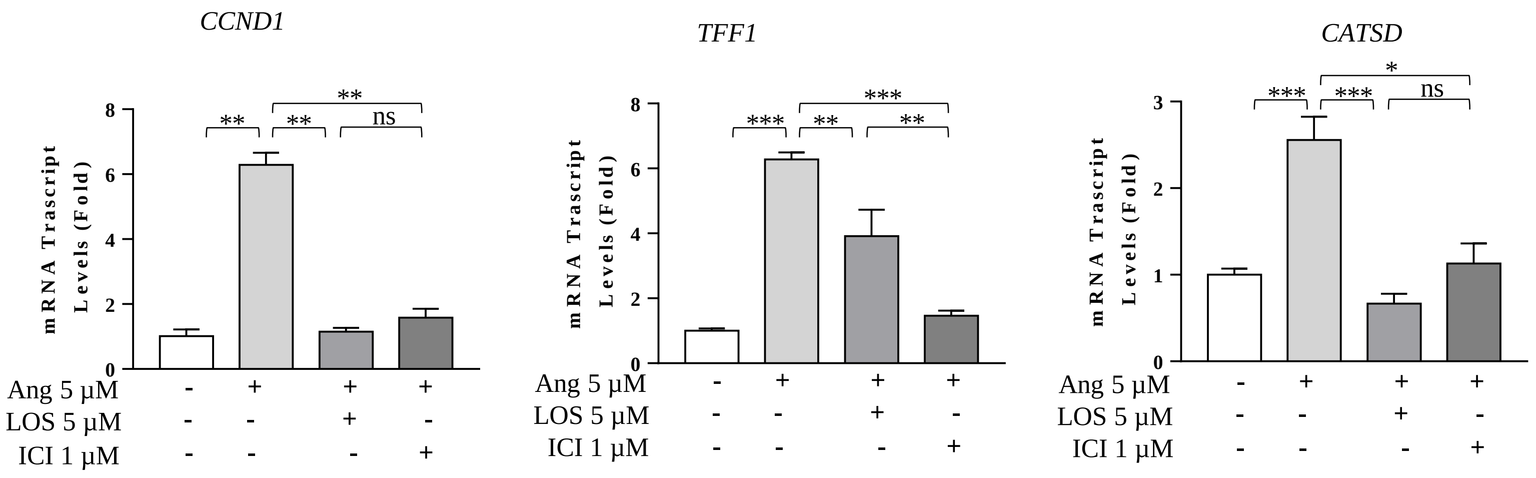

Figure S3. Effects of Angiotensin II receptor antagonist Losartan and ICI 182, 780 on ERα target genes expression in U-87 MG cells. Real-time RT-PCR for *CCND1*, *TFF1*, *CATSD* in U-87 MG cells. The cells were treated with vehicle (-), angiotensin II (Ang II, 5 µM) alone or in combination with losartan (LOS, 5 µM), angiotensin II (Ang II, 5 µM) in combination with ICI 182, 780 (ICI, 1 µM) for 24 hours. Data are expressed as means ± SD of three different experiments, each performed in triplicate. ***P < 0.001.
