# Supplementary material for: Novel Insights into the Antagonistic Effects of Losartan against Angiotensin II/AGTR1 Signaling in Glioblastoma Cells
Source: Cancers (Basel). 2021 Sep 10;13(18):4555. doi: 10.3390/cancers13184555 (PMC8469998; doi:10.3390/cancers13184555)
Supplement: Supplementary file 1 [file cancers-13-04555-s001.zip › Supplementary PDF/Figure S4.pdf]

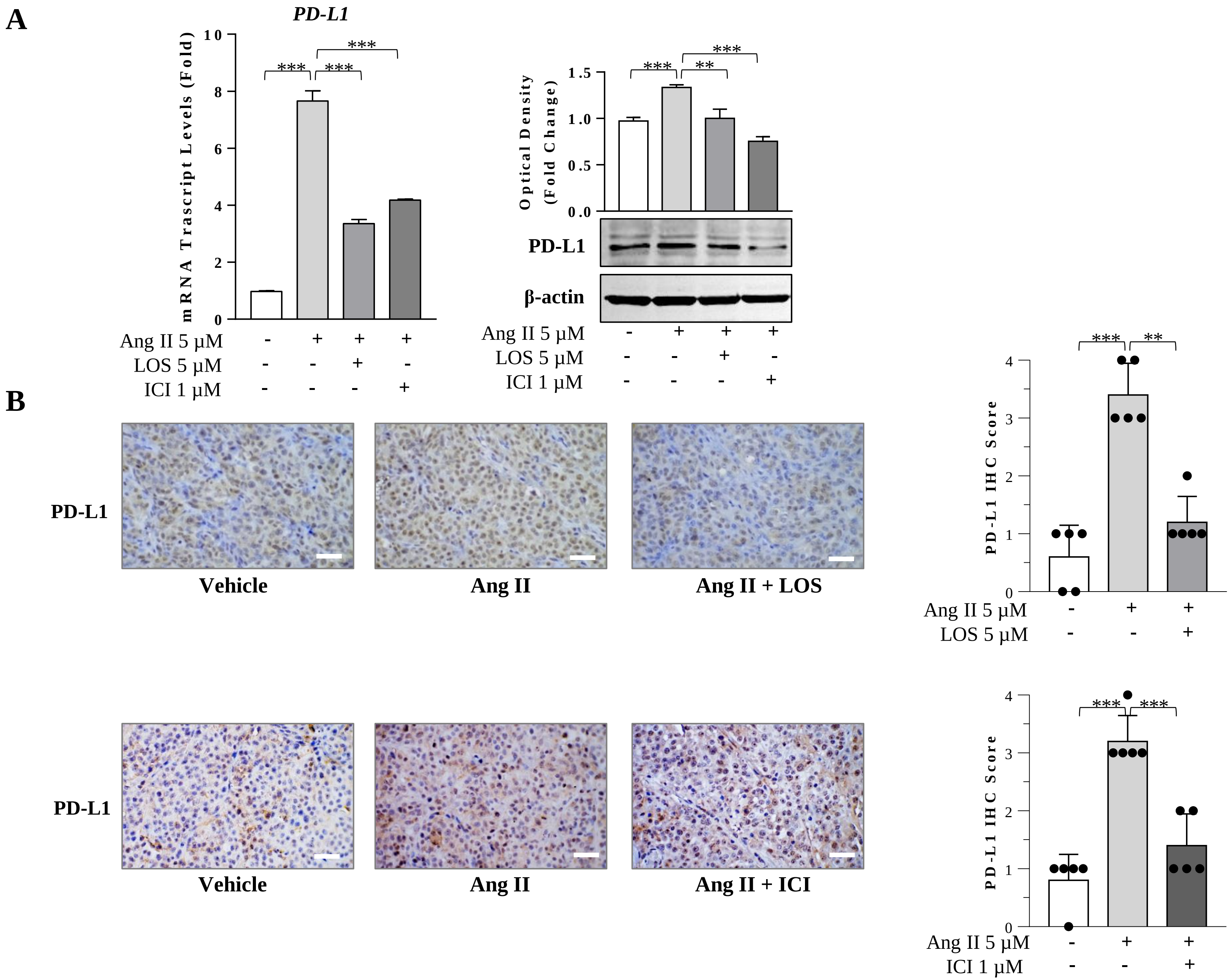

Figure S4. Effect of Angiotensin, Losartan and ICI on PD-L1 expression in glioblastoma cells, *in vitro* and *in vivo*. (A) PD-L1 mRNA and protein expression in U-87 MG treated with vehicle (-) and angiotensin II (ANG II, 5 µM) alone or in combination with losartan (LOS, 5 µM) or ICI 182, 782 (ICI, 1 µM) for 24 hours. The histograms represent the mean average ± S.D. of three separate experiments in which band intensities were evaluated in terms of optical density arbitrary unit and expressed as fold change over vehicle (-) for Ang II treatment, or for Ang II in combination with LOS or with ICI. β-actin was used as a control for equal loading and transfer. Data are expressed as means ± SD of three different experiments, each performed in triplicate.(B) Immunohistochemical analysis and relative score of PD-L1 in U-87 MG xenograft tumor sections upon Ang II treatment alone or in combination with losartan (LOS, 5 µM) or ICI 182, 782 (ICI, 1 µM). Scale bars = 12,5 µm. $^{**}P < 0.01$, and $^{***}P < 0.001$.
