# Supplementary material for: Novel Insights into the Antagonistic Effects of Losartan against Angiotensin II/AGTR1 Signaling in Glioblastoma Cells
Source: Cancers (Basel). 2021 Sep 10;13(18):4555. doi: 10.3390/cancers13184555 (PMC8469998; doi:10.3390/cancers13184555)
Supplement: Supplementary file 1 [file cancers-13-04555-s001.zip › Supplementary PDF/Figure S5.pdf]

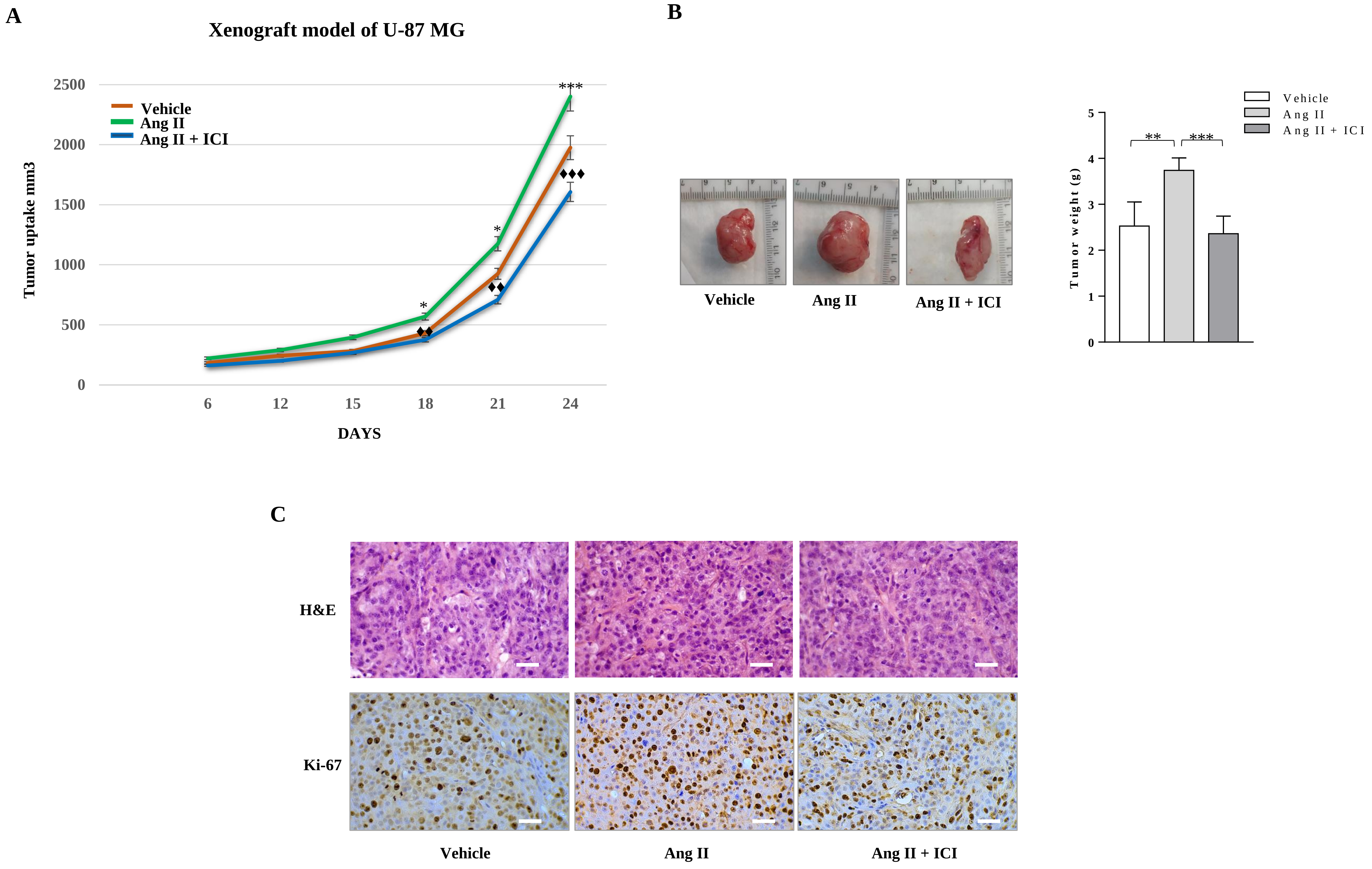

Figure S5: ICI 182, 780 downregulates Ang II-induced tumor growth of U-87MG xenograft. (A) U-87 MG cell were injected subcutaneously in female nude mice (five mice per group) and then treated with vehicle (-), Ang II alone or in combination with ICI. Relative tumor volume (RTV) was calculated by the following formula: RTV Z (Vx/V1), where Vx is the tumor volume on day x and V1 is the tumor volume at initiation of treatment (day 0). y axis: means ± SD of the RTV. (B) Representative tumor size and average tumor weight from each treatment group (C) Hematoxylin and eosin (H&E) staining and Ki-67 immunohistochemical analysis of tumor sections from vehicle (-), Ang II alone or in combination with ICI. Scale bars = 25 μm. *P < 0.05, **P < 0.01, and ***P < 0.001.
