# Supplementary material for: Novel Insights into the Antagonistic Effects of Losartan against Angiotensin II/AGTR1 Signaling in Glioblastoma Cells
Source: Cancers (Basel). 2021 Sep 10;13(18):4555. doi: 10.3390/cancers13184555 (PMC8469998; doi:10.3390/cancers13184555)
Supplement: Supplementary file 1 [file cancers-13-04555-s001.zip › Supplementary PDF/Figure S6.pdf]

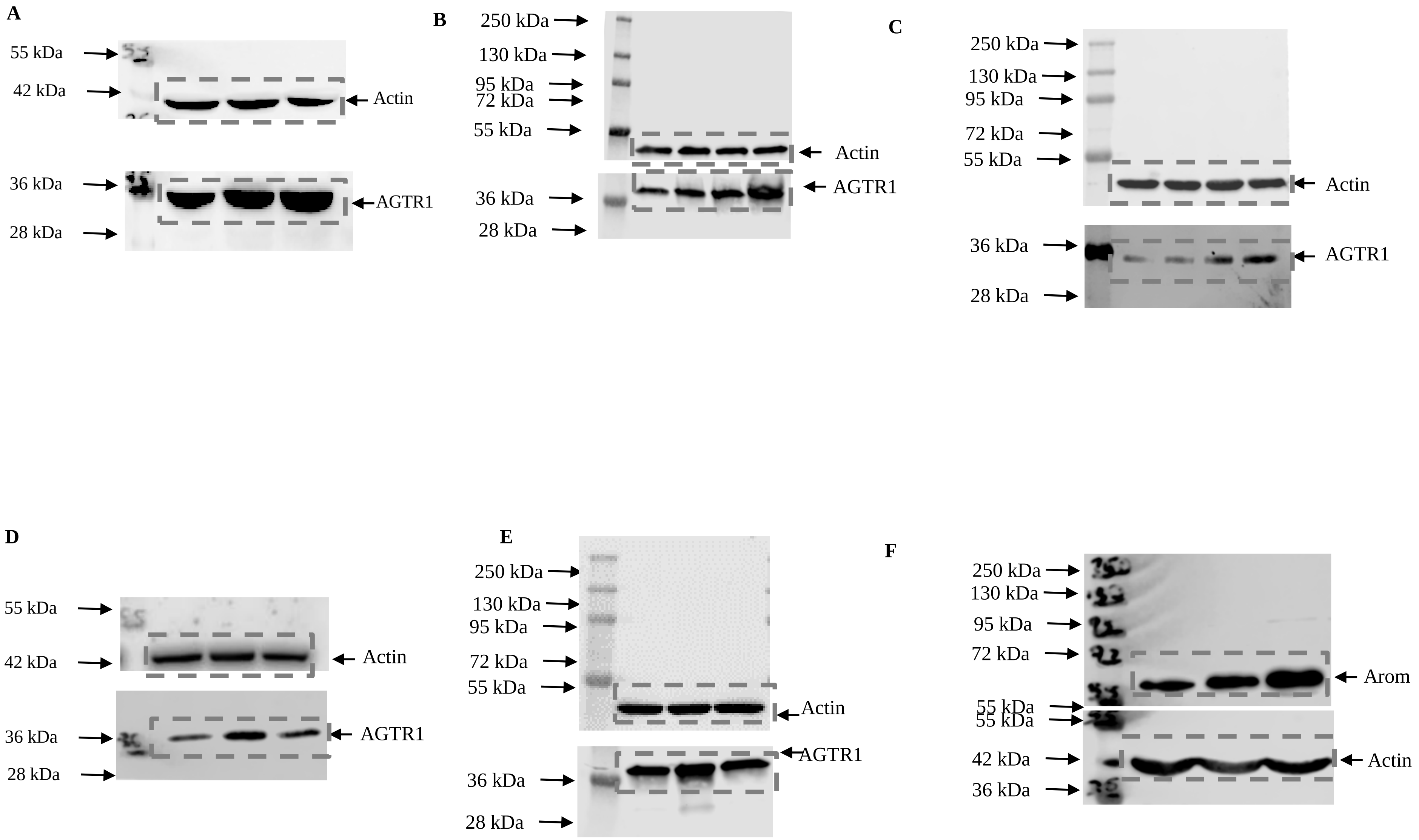

Figure S6. Uncropped western blots from primary figures are shown. (A) Figure 1B; (B), Figure 3A; (C) Figure 3B; (D), Figure 3C; (E), Figure 3D; (F), Figure 5B.
