# Supplementary material for: Novel Insights into the Antagonistic Effects of Losartan against Angiotensin II/AGTR1 Signaling in Glioblastoma Cells
Source: Cancers (Basel). 2021 Sep 10;13(18):4555. doi: 10.3390/cancers13184555 (PMC8469998; doi:10.3390/cancers13184555)
Supplement: Supplementary file 1 [file cancers-13-04555-s001.zip › Supplementary PDF/Supplementary Table 1_Panza et al., Cancers2021.pdf]

Supplementary Table S1. Oligonucleotide primers for Real Time PCR assays.

Gene Name	*Gene Symbol*	*Species*		*Primer Sequences*
Angiotensin II Receptor Type 1	*AGTR1*	*Human*	Forward<br>Reverse	5’-TCAGCCAGCGTCAGTTTCAA-3’<br>5’-CTACAAGCATTGTGCGTCGAAG-3’
Cytochrome P450 Family 19 Subfamily A Member 1	*CYP19A1*	*Human*	Forward<br>Reverse	5’-ACCCTTCTGCGTCGTGTCA-3’<br>5’-TCTGTGGAAATCCTGCGTCTT-3’
18s rRNA	*18s*	*Human*	Forward<br>Reverse	5’-CCCACTCCTCCACCTTTGAC-3’<br>5’-TGTTGCTGTAGCCAAATTCGTT-3’
Cyclin D1	*CCND1*	*Human*	Forward<br>Reverse	5’-ATGAACTACCTGGACCGCTT-3’<br>5’-CTTAGAGGCCACGAACATGC-3’
Trefoil Factor 1	*TFF1*	*Human*	Forward<br>Reverse	5’-GGGGTCCCCTGGTGCTTCTA-3’<br>5’-GCGTCAGGATGCAGGCAGA-3’
Cathepsin D	*CTSD*	*Human*	Forward<br>Reverse	5’-CCTCCATCCACTGCAAACTG-3’<br>5’-TTCACGTAGGTGCTGGACTT-3’
CD274	*CD274 Molecule*	*Human*	Forward<br>Reverse	5’-GGCATTTGCTGAACGCATT-3’<br>5’-ACAATTAGTGCAGCCAGGT-3’
Signal Transducer And Activator Of Transcription 3	*STAT-3*	*Human*	Forward<br>Reverse	5’-GTAGAAGGGTACAGTTCTCGG-3<br>5’-CCTAGGACACACATGCTCAC-3’
Jun Proto-Oncogene, AP-1 Transcription Factor Subunit	*c-JUN*	*Human*	Forward<br>Reverse	5’- CCCAAGTCAAACCTTACCTT-3’<br>5’- TAAAAGTCTCTCTACCTTCT-3’
