# Supplementary material for: Novel Insights into the Antagonistic Effects of Losartan against Angiotensin II/AGTR1 Signaling in Glioblastoma Cells
Source: Cancers (Basel). 2021 Sep 10;13(18):4555. doi: 10.3390/cancers13184555 (PMC8469998; doi:10.3390/cancers13184555)
Supplement: Supplementary file 1 [file cancers-13-04555-s001.zip › Supplementary PDF/Supplementary Table 2_Panza et al., Cancers2021.pdf]

Supplementary Table S2. Oligonucleotide primers for DAPA assays.

Gene Name	*Gene Symbol*	*Species*		*Primer Sequences*
Activator protein 1	*AP-1*	*Human*	Forward<br>Reverse	5'-TATTTCCTCCCTCAGAGGATGCC-3'<br>5'-GGCATCCTCTGAGGGAGGAAATA-3'
Mutant Activator protein 1	*mAP-1*	*Human*	Forward<br>Reverse	5'-TATTTCCTCaagtcaAGGATGCC-3'<br>5'-GGCATCCTCtgacttGAGGAAATA-3'
Signal Transducer And Activator Of Transcription 3	*STAT3*	*Human*	Forward<br>Reverse	5'-GTGTTTCCTGTGAAAGTTCC-3'<br>5'-GGAACTTTCACAGGAAACAC-3'
Mutant Signal Transducer And Activator Of Transcription 3	*mSTAT3*	*Human*	Forward<br>Reverse	5'-GTGTTTCcaatctAAGTTCC-3'<br>5'-GGAACTTagattGGAAACAC-3'
