# Supplementary material for: Novel Insights into the Antagonistic Effects of Losartan against Angiotensin II/AGTR1 Signaling in Glioblastoma Cells
Source: Cancers (Basel). 2021 Sep 10;13(18):4555. doi: 10.3390/cancers13184555 (PMC8469998; doi:10.3390/cancers13184555)
Supplement: Supplementary file 1 [file cancers-13-04555-s001.zip › Supplementary PDF/Supplementary Table 3_Panza et al., Cancers2021.pdf]

Immunostaining scores (Allred score median) of Ki-67 in U-87 MG xenograft

Vehicle	Ang II	Ang II+ ICI
6	*7*	*5**

Immunostained slides scores as follows: Total score= Proposition score + Intensity score (range 0-8)
*P< 0,005 (one-way ANOVA test) vehicle versus Treated

Supplementary Table 3. Immunostaining scores (Allred score median) of Ki-67 in U-87 MG xenograft.
