# Supplementary figures and images for: Novel Insights into the Antagonistic Effects of Losartan against Angiotensin II/AGTR1 Signaling in Glioblastoma Cells
Source: Cancers (Basel). 2021 Sep 10;13(18):4555. doi: 10.3390/cancers13184555 (PMC8469998; doi:10.3390/cancers13184555)

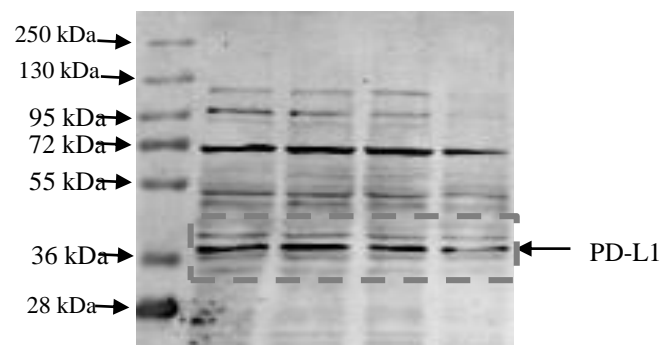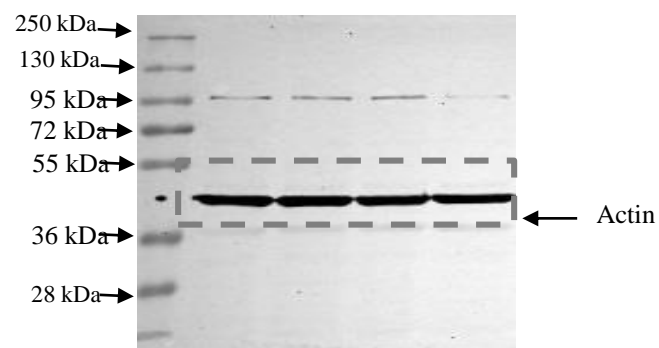

Figure S10. Uncropped western blots from primary Supplementary 4A are shown.

Supplement: Supplementary file 1 [file cancers-13-04555-s001.zip › Supplementary PDF/Figure S10.pdf]

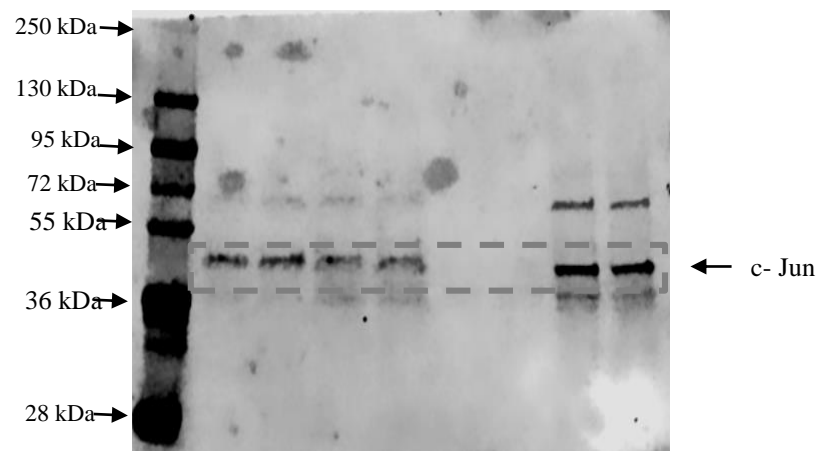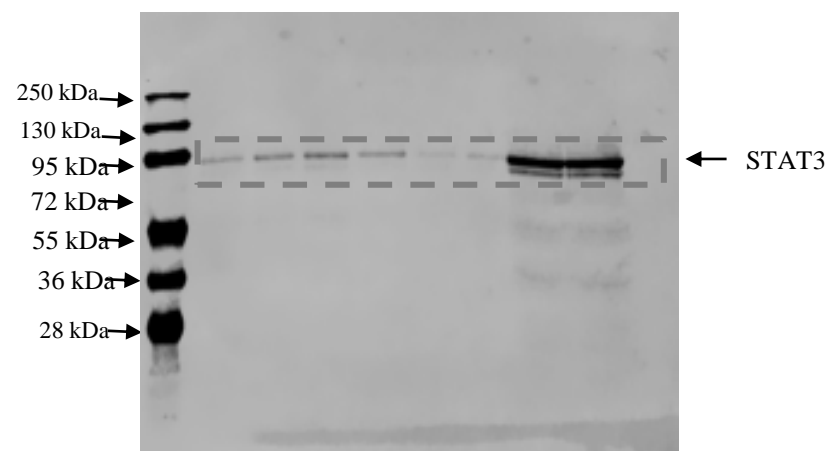

Figure S8. Uncropped western blots from figure 9C. are shown.

Supplement: Supplementary file 1 [file cancers-13-04555-s001.zip › Supplementary PDF/Figure S8.pdf]

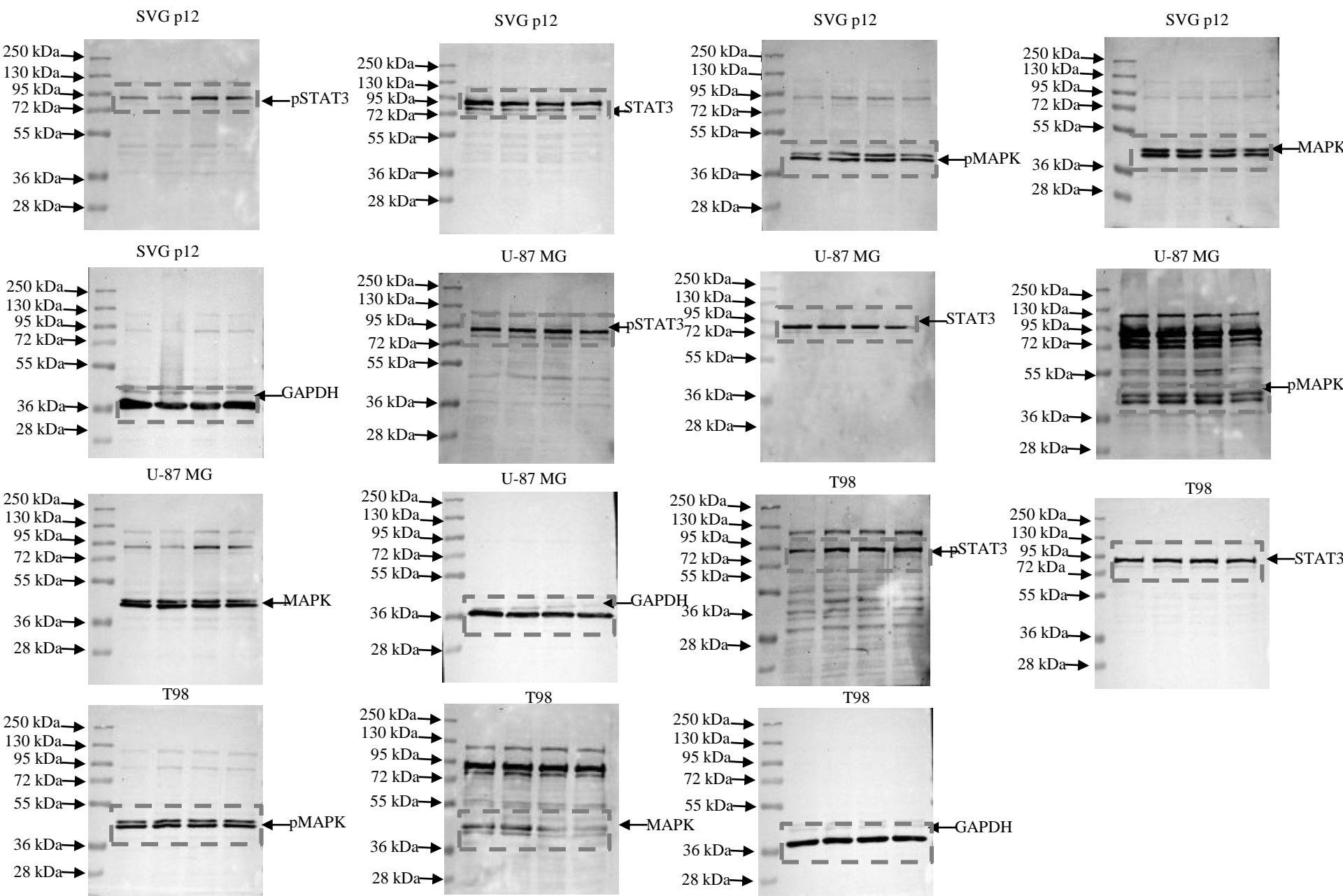

Figure S9. Uncropped western blots from Supplementary Figure 1 are shown.

Supplement: Supplementary file 1 [file cancers-13-04555-s001.zip › Supplementary PDF/Figure S9.pdf]
